# Evaluation of Duck Egg Hatching Characteristics with a Lightweight Multi-Target Detection Method

**DOI:** 10.3390/ani13071204

**Published:** 2023-03-30

**Authors:** Jiaxin Zhou, Youfu Liu, Shengjie Zhou, Miaobin Chen, Deqin Xiao

**Affiliations:** 1 College of Mathematics Informatics, South China Agricultural University, Guangzhou 510225, China; 2 Key Laboratory of Smart Agricultural Technology in Tropical South China, Ministry of Agriculture and Rural Affairs, Guangzhou 510225, China

**Keywords:** machine vision, deep learning, duck eggs, non-destructive testing

## Abstract

**Simple Summary:**

The aim of this study was to improve the hatching efficiency of duck eggs by automatically assessing the hatching characteristics of early hatching eggs. The timely and accurate detection of fertile and infertile eggs is an important research topic in the breeder egg hatching industry. Detecting infertile eggs early in the hatching process not only improves the hatching efficiency of duck eggs, but also brings benefits to hatching companies. In recent years, the rapid development of deep learning and computer vision technology has brought us new ideas. We propose a lightweight multi-target detection method based on deep learning to evaluate the hatching characteristics of duck eggs. The results show that the method could meet the requirements for accuracy and real-time detection in industrial production.

**Abstract:**

Since it is difficult to accurately identify the fertilization and infertility status of multiple duck eggs on an incubation tray, and due to the lack of easy-to-deploy detection models, a novel lightweight detection architecture (LDA) based on the YOLOX-Tiny framework is proposed in this paper to identify sterile duck eggs with the aim of reducing model deployment requirements and improving detection accuracy. Specifically, the method acquires duck egg images through an acquisition device and augments the dataset using rotation, symmetry, and contrast enhancement methods. Then, the traditional convolution is replaced by a depth-wise separable convolution with a smaller number of parameters, while a new CSP structure and backbone network structure are used to reduce the number of parameters of the model. Finally, to improve the accuracy of the network, the method includes an attention mechanism after the backbone network and uses the cosine annealing algorithm in training. An experiment was conducted on 2111 duck eggs, and 6488 duck egg images were obtained after data augmentation. In the test set of 326 duck egg images, the mean average precision (mAP) of the method in this paper was 99.74%, which was better than the 94.92% of the YOLOX-Tiny network before improvement, and better than the reported prediction accuracy of 92.06%. The number of model parameters was only 1.93 M, which was better than the 5.03 M of the YOLOX-Tiny network. Further, by analyzing the concurrent detection of single 3 × 5, 5 × 7 and 7 × 9 grids, the algorithm achieved a single detection number of 7 × 9 = 63 eggs. The method proposed in this paper significantly improves the efficiency and detection accuracy of single-step detection of breeder duck eggs, reduces the network size, and provides a suitable method for identifying sterile duck eggs on hatching egg trays. Therefore, the method has good application prospects.

## 1. Introduction

For the duck egg hatching industry, the hatching rate of duck eggs has a direct impact on the economic efficiency of a plant. The duck egg incubation process takes about 28 days and requires the temperature to be maintained at 37 °C to 38 °C, with high requirements for ambient temperature and humidity. In hatchery plants, a large amount of energy is consumed to ensure stable and concentrated hatching conditions. The fertility rate of duck eggs under natural conditions is around 85% [1]. There is a general lack of early activity detection equipment in the duck egg incubation process. This leads to a large proportion of infertile duck eggs being included in the centralized hatching process, which can result in a great waste of resources. At the same time, infertile duck eggs may deteriorate during incubation, releasing harmful gases that can contaminate already fertilized breeding eggs and affect the overall hatching effect [2]. In addition, the incubation environment can change during the identification of infertile duck eggs. This can lead to a drop in egg temperature, which can affect the hatching rate of the eggs. therefore, a high level of real-time performance is required in the equipment for identifying fertilized and infertile eggs. Most of the devices in the production environment are embedded devices and do not have a lot of memory. Therefore, a fast, accurate and lightweight method is needed to detect fertile and infertile eggs.

Identifying the hatching characteristics of duck eggs depends mainly on the internal characteristics of the eggs, for example, the thermal infrared and visible light characteristics inside the duck egg. When a thermal infrared detector is used to detect an incubating duck egg, the difference in the temperature change profile reflects the difference between fertile and infertile eggs. When duck eggs are exposed to darkness after 7 days of incubation, the spots and blood vessels inside the eggs can be observed by shining candlelight on fertile eggs. For infertile eggs, the intra-egg features are essentially the same as before hatching.

In the hatching industry, duck eggs are usually selected after seven days of incubation, and skilled workers use candlelight to distinguish fertile eggs from infertile eggs [3]. However, this method has problems such as slow detection speed, low efficiency and the subjective influence of workers, which cannot well meet the current automated production requirements.

Recently, the literature [4] has proposed a deep learning-based approach that combines migration learning strategies with neural networks. The classification problem in small-scale datasets of hatching eggs was successfully solved. The authors of [5] developed a method for detecting well-developed breeder eggs using machine vision. The images were enhanced using histogram stretching and parameters were calculated to describe the histogram shape of the resulting images. Finally fertilized and sterile eggs were distinguished based on the histogram shape parameters. Another study [6] trained neural networks to identify shape differences in the grey-scale histograms of fertilized and sterile egg images at early hatching stages. Other researchers have also attempted to identify sterile eggs and monitor embryonic development using NIR hyperspectral imaging [7], thermal imaging [8,9,10] and VIS/NIR spectroscopy [11,12,13]. All these methods yielded good results, but they were specific to individual eggs and were difficult to apply in practical production. A method for evaluating the hatching characteristics of multiple duck eggs on incubation trays was proposed in the literature [14]. Based on image analysis of duck eggs incubated for 7 days, a region of interest mask was created and a vector of image feature parameters with four features was extracted. A discriminative model for different hatching periods was developed by machine learning methods to solve the problem of difficult segmentation of duck egg clusters, and the average recognition accuracy of the method reached 92.06%. However, there is still room for further optimization in detection accuracy. The YOLO (You Only Look Once) family of algorithms belongs to the One-Stage family of algorithms and does not have a process for generating bounding boxes [15], which can guarantee the real-time nature of the model and thus are widely used in the agricultural field [16,17,18,19,20,21,22,23]. Because embedded devices often do not have a large memory, the smaller the model, the easier it is to deploy to embedded devices. Therefore, to meet the requirements for detection speed, model size and number of detections in real production, this paper proposes a new lightweight detection architecture (LDA) based on the YOLOX-Tiny framework to identify the hatching characteristics of duck eggs with 5 days hatching. The method reduces the size of the model while ensuring the detection accuracy and detection speed of the model. The main contributions of this paper are as follows: (1) constructing an image acquisition system for multiple duck eggs on an incubation tray; (2) introducing an attention mechanism to improve the detection of infertile duck eggs; and (3) proposing a novel lightweight LDA method to identify the hatching characteristics of duck eggs, and performing extensive experiments showing that the method has a good balance between detection accuracy and model size. In the following sections, we first introduce the data acquisition and augmentation pre-processing scheme, then discuss the optimization strategy mechanism of the LDA algorithm, and finally conduct experimental validation and analysis.

## 2. Materials and Methods

### 2.1. Materials Preparation

#### 2.1.1. Samples Preparation and Collection Platform

The duck eggs named Tianlu shelduck N107 were used in this study and were obtained from the Wens Foodstuff Group. The total number of eggs was 2111, which included 840 infertile eggs and 1271 fertile eggs.

We disinfected the duck eggs by wiping them with alcohol and gauze. Once the surface of the duck egg was free of dirt, the infertile eggs were numbered together with the fertile eggs using an oil-based pen and placed in a numbered industrial egg tray with a size of 7 × 9. The egg trays were labelled with capital letters and the breeding duck eggs were numbered and placed in order. The duck eggs were placed vertically in the egg tray with the gas chamber facing upward, and the number was written at the end away from the gas chamber to avoid contaminating the experimental data. The treated duck eggs in the egg trays were placed in a smart incubator, and data were collected every 24 h. The images of the fifth day were selected to create the dataset. The ambient temperature at the time of data collection was controlled between 19 °C and 21 °C. Finally, the eggs were broken after 20 d of incubation. Eggs with developing and forming ducklings were judged as fertile eggs, and eggs that were undeveloped or had blood rings were judged as infertile eggs. In other words, eggs were considered fertile when the outline of a duckling was observed to develop. The post-developmental mortality of ducklings is not considered in this paper.

The egg image acquisition system consisted of an industrial camera, duck egg tray, candlelight system, computer and dark box. The camera model was DW1200; the height of the camera from the ground was 70 CM, and the center point of the camera was perpendicular to the egg tray. The candlelight system contained seven rows of nine columns, making of a total of 63 LED light sources, each with a power of 5 W. The computer model was D14U (this computer was only used for acquiring the experimental data). The resolution of the acquired images was 1092 × 1080, and the original images were saved in JPEG format. 

**Figure 1:**
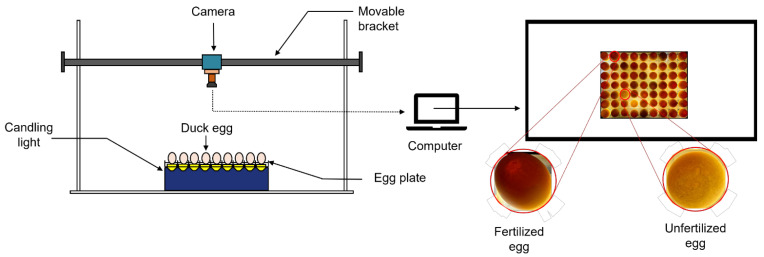
Scheme of the image acquisition system.

The image acquisition system is shown in Figure 1.

#### 2.1.2. Data Augmentation

**Figure 2:**
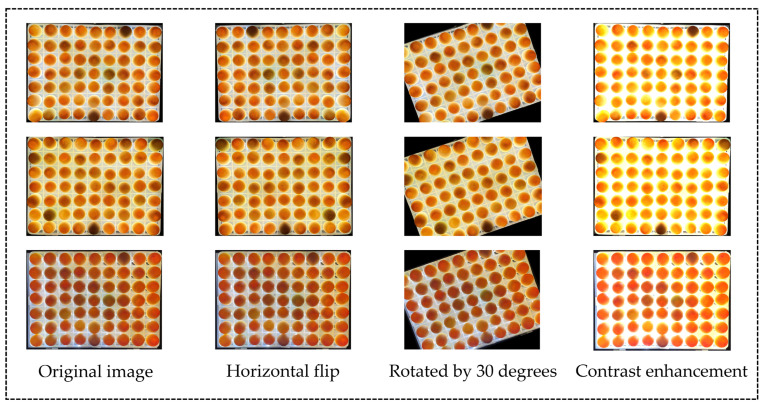
Examples of enhanced images used to simulate problems in analysis.

When identifying the hatching state of duck eggs, the recognition effect was mainly affected by the brightness of light, shooting angle and other factors. Therefore, augmentation techniques such as rotation, reversing the symmetry and changing the contrast of the original images were used to augment the experimental dataset. The environmental conditions under different angles in the real production environment were simulated by changing the angle of the original image; the image contrast was enhanced to simulate the problem of light source interference in the production environment. The data augmentation effects are shown in Figure 2.

The dataset after data augmentation is shown in Table 1. Prior to training, the acquired raw images needed to be manually annotated. Fertile and infertile eggs were labeled in all processed images using LabelImg (A target detection dataset annotation tool). After labeling, the software automatically generated an XML file corresponding to the names of the original images. It contained the location information of all duck eggs and finally stored the data as a PASCAL (Pattern analysis, statistical modelling, and computational learning) VOC (Visual object classes) dataset for the training of the algorithm in this paper.

### 2.2. LDA Algorithm Optimization Mechanism

**Figure 3:**
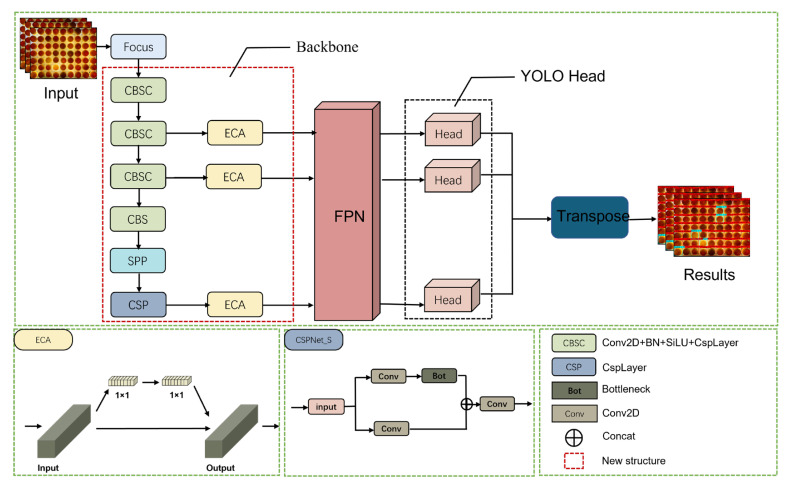
LDA structure.

The YOLO family of algorithms is a classical algorithm in the field of target detection. This series of algorithms is popular among researchers because of its good real-time performance [24]. For the problem of identifying infertile duck eggs on egg trays in early hatching, this paper proposes an improved LDA algorithm based on YOLOX-Tiny. The algorithm structure consists of 3 parts: a backbone feature extraction network, feature pyramid networks (FPN) and a detection network [25,26,27]. The backbone feature extraction network uses the CSPDarknet (CSPDarknet consists of CSPNet, which stands for Cross Stage Partial Network, and Darknet, which is an open source neural network framework) to convolve the image to extract features. The spatial pyramid pooling (SPP) layer is used at the end of the backbone network to improve the perceptual field of the backbone network. FPN is used to enhance feature extraction by fusing the three features extracted from the backbone network as a way to fuse feature information at different scales. In addition, an anchor-free structure [27] is also used to solve the problem of scale detection. The LDA structure is shown in Figure 3.

#### 2.2.1. Model Lightweighting Strategy

Analyzing the YOLOX network, its basic composition is Conv + BN + Silu, i.e., one convolution followed by a batch normalization and then an activation function. Therefore, a large number of convolutions are included in the whole YOLOX network. Depth-wise separable convolutions (DWConv) consist mainly of Depthwise convolutions and Pointwise convolutions. Compared with a normal convolution, the number of parameters in a deep separable convolution is approximately one third of that of a normal convolution. The use of depth-wise separable convolutions instead of convolutions can save a large number of parameters [28]. In a neural network, a smaller number of parameters means a smaller model size and less computation of the model during the computation. The covariance equations of the two convolution methods are as follows:

(1) $$c=m\times k^{2}\times p^{2}\times N$$

(2) $$Sd=m\times k^{2}\times p^{2}+m\times p^{2}\times N$$

(3) $$\frac{Sd}{Sc}=\frac{1}{N}+\frac{1}{k^{2}}$$

where *Sc* is the number of normal convolutional parameters, *Sd* is the number of depth-separable convolutional parameters, *m* is the number of input image channels, *k* is the convolutional kernel size, *p* is the number of convolutional kernel slides, and *N* is the number of convolutional kernels.

According to the above equations, it is clear that replacing the convolution with a depth-wise separable convolution can significantly reduce the number of parameters of the model. The number of network parameters as well as the floating point operations (FLOPs) after replacing the convolution in the network with the depth-wise separable convolution are shown in Table 2.

**Figure 4:**
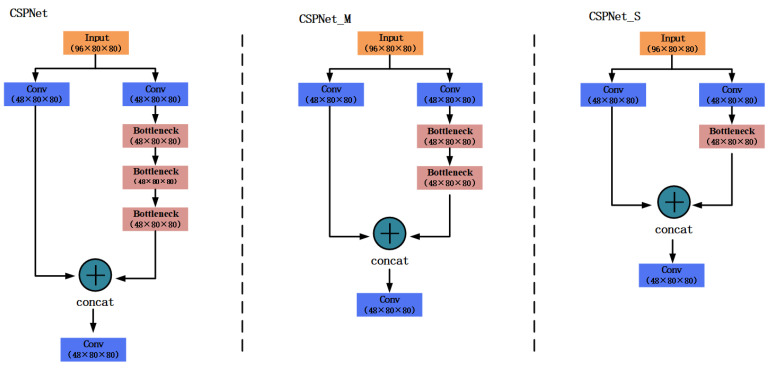
Three CSPNet Structures.

Although the parameters of the network can be greatly reduced by using a depth-wise separable convolution, the network structure itself is not optimized. Therefore, the network structure can also be optimized to reduce the number of parameters. Notice that in the duck egg dataset, there are only two categories, i.e., sterile as well as fertile eggs, and the number of categories is greatly reduced compared with the other datasets. The fewer the categories, the lower the difficulty of network learning. Therefore, a simple network structure is sufficient for learning duck egg features. It was observed that the YOLOX backbone network consists of four CSPNets, so the optimization of the network can be considered in two directions. On the one hand, we could consider reducing the number of CSPNet, and on the other hand, we could consider reducing the complexity of CSPNet [29]. Since there are only four CSPNets in total, the number of CSPNets must be reduced very carefully, so we focused on reducing the complexity of CSPNets. Notice that the original CSPNet divided each feature matrix into two parts, one part left untouched and the other convolved with the residual blocks (Bottleneck). This dual-path structure enhances the learning capability of the network and ensures accuracy, while being lightweight so it cannot be altered. The role of the residual blocks is to extract information at different levels. Therefore, we could reduce the number of residual blocks appropriately without affecting the performance of the model. We designed two new network structures, CSPNet_M and CSPNet_S, to replace the original CSPNet. The structure of these three networks is shown in Figure 4. In the CSPNet_M network, the feature map is first convolved by a 3 × 3 convolution kernel. The number of output channels is reduced to half the original and then entered a two-path network. The first path is left unprocessed, and the other path is convolved with the residual blocks twice. Then the convolution results are stacked with the feature maps on the first path to complete the channel expansion. The stacked result is output after a 3 × 3 convolution. CSPNet_S was compared with CSPNet_M by removing a residual block on top of CSPNet_M. To compare the complexity of the three networks, we used floating point operations (FLOPs) and the number of parameters to indicate the size of the modules. We performed the calculation with an input feature layer size of 80 × 80 and the number of channels was 96, as shown in Table 3, where Conv means the network consists of a convolution and DWConv means the network consists of a depth-wise separable convolution. As can be seen in Table 3, the number of parameters of CSPNet_M is reduced by 15.3% and FLOPs by 15.5% compared with CSPNet for the network based on depth-wise separable convolution, and CSPNet_S was reduced by 18.0% and FLOPs by 18.4% compared with CSPNet_M.

**Figure 5:**
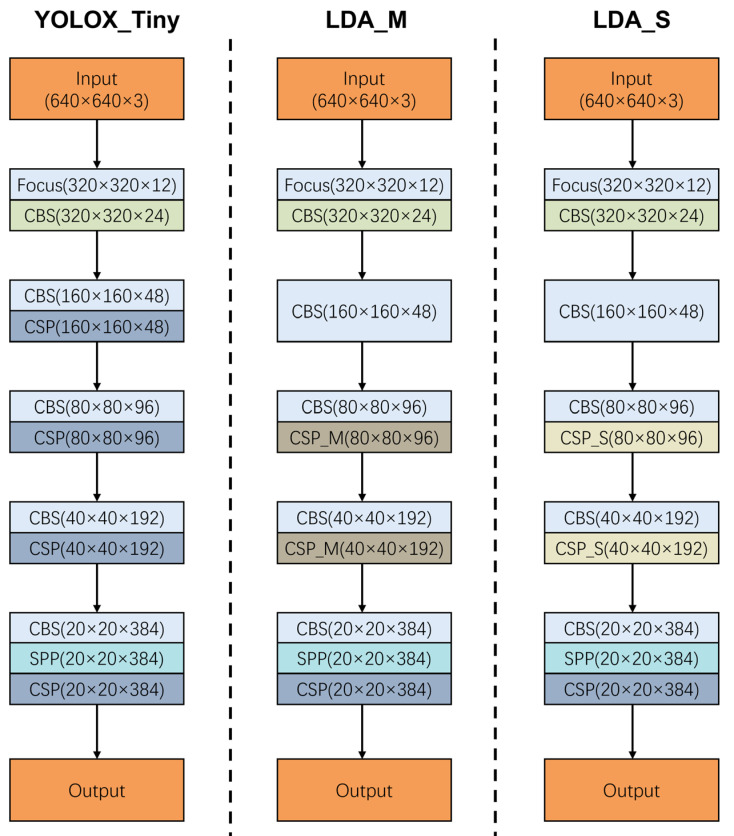
Three backbone network structures.

The backbone network is the process of feature extraction, image size compression, and channel number expansion for images. Since the YOLOX backbone network consists of four CSPNets, the number is small and the outputs of the last three CSPNets are used as inputs to the FPN structure. Therefore, we removed the first CSP structure and used a common convolution operation for the expansion of the number of channels. Meanwhile, based on the proposed CSPNet_M and CSPNet_S, we designed two new backbone networks named LDA_M and LDA_S. The first CSPNet structure is removed from both LDA_M and LDA_S. In addition, in LDA_M, the second and third CSPNet structures are replaced with CSPNet_M, keeping the last CSPNet structure unchanged. In LDA_S, the second and third CSPNet structures are replaced with CSPNet_S, keeping the last CSPNet structure unchanged. The structure of the three backbone networks is shown in Figure 5. The model sizes of the three backbone networks are shown in Table 4. It can be seen that in the proposed two new network structures, the number of network parameters as well as the FLOPs are gradually reduced. With depth-wise separable convolution as the base convolution, the number of parameters of network LDA_S is reduced by 5.6% compared with the original YOLOX_Tiny and by 2.5% compared with network LDA_M. The FLOPs of network LDA_S are 23.0% less than those of network YOLOX_Tiny and 16.7% less than those of network LDA_M. The structure of network LDA_S is the simplest with the least number of parameters and FLOPs, so the network LDA_S is referred to as LDA in this paper.

#### 2.2.2. Attention Mechanisms Based on ECA Structures

**Figure 6:**
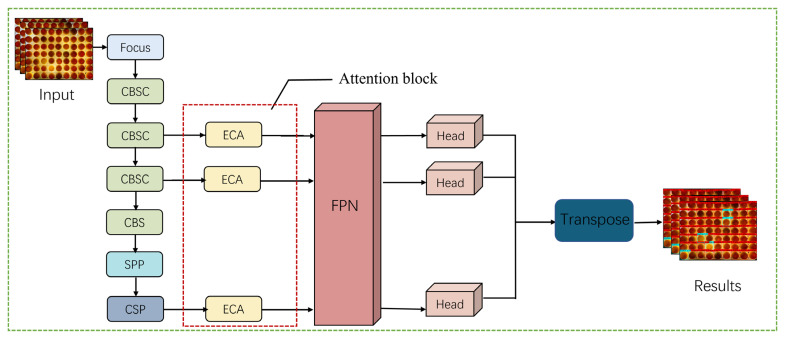
Network structure after adding attention mechanism.

Due to the use of a large number of depth-wise separable convolutions and lightweighting measures such as optimizing the network structure, there are significant reductions in network parameters and computation while at the same time reducing accuracy. In recent years, attention mechanisms have been widely used to improve the accuracy of deep learning methods [30]. Therefore, to address the problem of reduced accuracy after network lightweighting, we added an attention mechanism to enhance the feature extraction capability of the detection network as a way to improve the accuracy of the network. In this study, in the egg activity detection network, an attention block is added after the three outputs of the backbone network. By extracting cross-channel information, the feature information of the duck egg is emphasized and the background information is weakened, thus achieving an improvement in the detection performance of the network. The structure of the network with the added attention mechanism is shown in Figure 6.

The attention module consists of three parts: input, cross-channel information extraction, and output. Most existing approaches are dedicated to developing more complex attention modules to achieve better performance, which inevitably increases the complexity of the model [31]. To overcome the tension between performance and complexity, the attention mechanism module used in this paper is ECANet [31]. ECANet first performed a global average pooling of the incoming feature maps without dimensionality reduction, and subsequently performed attention extraction using a 1 × 1 convolution of the neighboring 3 channels of each channel. In addition, ECANet contains a method for adaptively selecting the size of the one-dimensional convolutional kernel to determine the coverage of local cross-channel interactions. Thus, ECANet allows the model to focus more on detailed information relating to fertile versus infertile eggs, which is useful for improving the model’s detection performance.

## 3. Results and Discussion

### 3.1. Model Training and Application Based on Cosine Annealing Learning Rate 

#### 3.1.1. Cosine Annealing Algorithm

During the training of deep learning networks, the gradient descent algorithm is often used to make the network converge to the optimal solution, keeping the loss value of the loss function as close as possible to the global minimum rather than the local minimum. In the deep network training process, it is easy for the network to fall into the “saddle plane”, i.e., the point where the gradient is 0. Since the gradient is 0, the model cannot converge further, and it easily falls into local minima, causing the model to stop updating. Cosine annealing algorithm with initial learning rate as the maximum learning rate. It decreases and then increases in one cycle. The reason for repeating this process is that the learning rate continues to decrease as the model is trained more and more. After a period of training, the model is likely to fall into a “saddle plane” with a small gradient, so the learning rate was increased to the initial value in the hope that a larger learning rate would allow the model to break out of the “saddle plane”.

The principle of the cosine annealing algorithm is as follows:

(4) $$\eta_{t}=\eta_{min}^{i}+\frac{1}{2}\left(\eta_{max}^{i}-\eta_{min}^{i}\right)\left(1+cos\left(\frac{T_{cur}}{T_{i}}\pi \right)\right)\;$$

where $\eta_{min}^{i}$ is the minimum learning rate, $\eta_{max}^{i}$ is the maximum learning rate, $T_{cur}$ is the number of cycles currently being learned, and $T_{i}$ is the total number of cycles in the current operating environment.

#### 3.1.2. Model Training

The training of the model is essentially a process of approximating the loss value of the model loss function to the optimal solution, i.e., the predicted value of the model converges to the true value. The parameters of the network model training are as follows: the image size is 640 × 640 pixels, the batch size is set to 2, the initial learning rate is 0.001, the weight decay is 0.0005, the momentum factor is 0.9, and the cosine annealing algorithm is used. The training parameters for this paper are shown in Table 5.

One of the metrics that measures the effectiveness of model training is the loss value. The convergence of the model is independent of the loss value. However, all other things being equal, the smaller the loss value after convergence, the better the model is trained. When the loss value of the model is basically unchanged, it means that the model converges. In contrast, in the training of the LDA network without using the cosine annealing algorithm, the iterations leveled off after 300 iterations and the loss value converged to about 30, proving that although the loss value converged, the model clearly fell into local minima in training and converged to near the local minima. This was because in the model without the cosine annealing algorithm, the learning rate decays gradually to very small values as the number of iterations increases. At this point, if the local minima are encountered, it is difficult for the model to jump out of the “saddle plane”.

However, even using the cosine annealing algorithm, the loss value of the model eventually converged to only about 1.8. It hovered around the global minimum and could not approach the global minimum further. This had a negative impact on the training of the whole model. Therefore, in order to further converge, the cosine annealing algorithm was turned off in the last seventy epochs of the model training. After turning off the cosine annealing algorithm, the model loss value finally converged to about 1.35. This was better than 1.8 without turning off the cosine annealing algorithm, and 25% lower than the loss value without turning off the cosine annealing algorithm.

### 3.2. Experimental Environment and Evaluation Index

The operating platform for the experimental algorithm in this paper was a desktop computer with an Intel Core I5-11400 processor with a default frequency of 2.60 GHz, 16 GB of RAM, and a NVIDIA GeForce RTX 3070 video card with 8 GB of video memory. The development environment was Windows Professional, Python 3.7.11, anaconda 4.10.1, and CUDA version 11.4. The deep learning framework was pytorch-GPU-1.7.1. The more popular technical metrics for evaluating the performance of deep learning network models are Precision-Recall curve (P-R curve for short), AP (detection accuracy), mAP (mean value of AP values under all categories), accuracy (Acc), detection speed, and the number of model parameters. In this paper, six metrics, AP, mAP, detection speed, number of model parameters, and floating point computation, are used to compare the advantages and disadvantages of the networks. The AP value is the area enclosed by the P-R curve and the coordinate axis. mAP is the average value of all AP sums. The calculation of P is detailed in Equation (5), and the calculation of R is shown in Equation (6). Accuracy is the ratio of the number of correct predictions to the number of all samples in the prediction sample.

(5) $$R=\frac{T_{P}}{T_{P}+F_{N}}$$

(6) $$P=\frac{T_{P}}{T_{P}+F_{P}}\;$$

*TP*, *FP* and *FN* are true positives, false positives and false negatives, respectively.

### 3.3. Performance Analysis of LDA Model Optimization Strategys

In order to evaluate the effectiveness of the model after lightweighting, we conducted ablation experiments to verify the performance of different optimization methods. The optimization of the models in this paper are based on YOLOX-Tiny, so we used the YOLOX-Tiny model as the benchmark for the ablation experiments.

As shown in Table 6 above, when we used deep separable convolution, the number of parameters of YOLOX-Tiny decreased dramatically. While the mAP decreased by only 0.52%, the LDA_M network was improved from YOLOX-Tiny + DW. With the optimization of the backbone network, the mAP of LDA_M increased by 3.68%, the number of parameters decreased by 0.031 M, and the FLOPs decreased by 0.477 G. Further optimization of the structure of the LDA_M network yields LDA_S, at which point the mAP of the model continued to rise, reaching 99.58%, an increase of 1.46% over the mAP of LDA_M. The number of parameters decreased by 0.025 M, and FLOPs decreased by 0.138. After adding the ECA attention mechanism, the mAP of the model rose, while the size of the model hardly increased. Taking LDA_S as an example, after adding the ECA module, the mAP of LDA_S increased by 0.16%, but the FLOPs only increased by 0.003 G. In addition, the LDA_S with the ECA added was compared with the YOLOX-Tiny without the changes. It can be clearly seen that the mAP improved by 4.82%, indicating that the improvements made in this study significantly improved the detection accuracy of the model. The number of parameters was reduced from 5.033 M to 1.935 M, a decrease of 61.55%, and the FLOPs were reduced from 15.233 G to 4.667 G, a decrease of 69.36%. The results show that the detection accuracy of the model can be guaranteed despite the significant reduction in model size. Finally, we carried out a one-way ANOVA for mAP with different improvement methods at the 5% probability-based level, as shown in Table 7 below. Based on these results, it can be seen that there were significant differences in the results for the different improvement methods. There were also significant differences between the results of the original model (YOLOX-Tiny) and the optimal model (LDA_S + ECA). Considering the factors of model size and accuracy, it is clear that LDA_S + ECA is the best network. The results in Table 6 and Table 7 show that the improved solution proposed in this study is effective.

### 3.4. Comparative Analysis of Model Lightweight Performance

In this experiment, the test results of 326 duck eggs after 5 days of incubation were counted using different target detection networks (YOLOv4-Tiny, YOLOv5-S, YOLOX-Tiny, LDA_M, YOLOX-Nano, LDA). Five evaluation metrics including AP, mAP, detection speed, number of model parameters and FLOPs were included. The statistical results are shown in Table 8.

**Figure 7:**
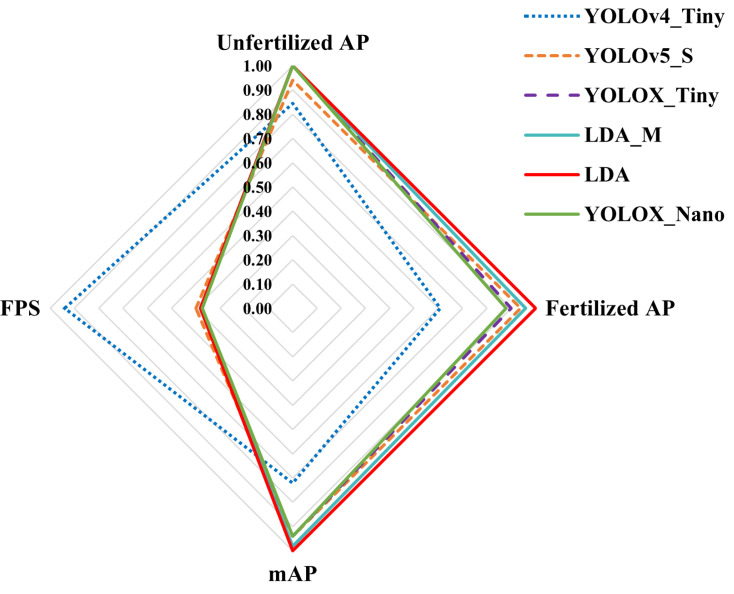
Accuracy of using different methods to detect the fertility status of breeding eggs.

The duck egg dataset used in this study had only two types with uniform image backgrounds. Compared with scenes with complex backgrounds, the recognition difficulty was low. Therefore, the requirements for depth and width of the network were not particularly high, and a lightweight network structure could be designed to meet the requirements of detection accuracy and detection speed. Based on the YOLOX-Tiny network, we introduced depth-wise separable convolution instead of convolution, modified the backbone network structure to reduce the number of network parameters and simplified the network structure. The accuracy of the network was further improved by using the attention mechanism ECANet. We retained the network with the highest detection accuracy and excellent detection speed, namely “LDA_S + ECA”, referred to as “LDA”. In this subsection, we refer to “LAD_M + ECA” as “LAD_M”. As can be seen from Table 6, although the YOLOX-Nano network had the lowest number of participants, the overall detection effect of the YOLOX-Nano network was not good due to the limitation of network size and network depth. Although the detection effect was better than that of the YOLOv4-Tiny network, the overall accuracy performance was slightly poor, and the AP for infertile eggs was less than 90%, which is not sufficient to be used in the actual production environment. Although YOLOv4-Tiny had the fastest inspection speed, it also could not meet the actual production needs due to the low inspection accuracy. The mAP of YOLOX-Tiny was 94.92%, which was a good performance. However, its number of parameters was relatively large, and the mAP was not optimal. Observing the networks LDA_M and LDA, although the number of parameters of LDA_M was larger than LDA, the detection accuracy of its network was instead 1.44% lower than LDA due to the problem of unstable gradients and network degradation brought by the deep network. Finally, we conducted a one-way ANOVA on the mAP of the different models on a 5% probability basis, as shown in Table 9 below. Based on the results in Table 9, it can be seen that the results of LDA were significantly different from those of the other models. Therefore, the combined results of Table 8 and Table 9 show that the LDA network had better generalization performance and the model had better reliability. To show the detection performance of the model more intuitively, the data in Table 8 were normalized and then subjected to radar plot analysis, and the results are shown in Figure 7.

### 3.5. Analysis of Different Feature Network Models for Multi-Target Detection Capability

**Figure 8:**
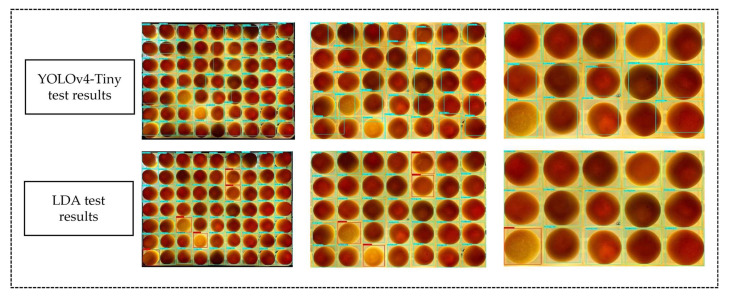
Detection results.

In the actual production environment of breeder duck egg hatching, different manufacturers may have different duck egg trays. The size of the egg tray and the distance of the camera could affect the size of individual breeding eggs in the picture to be tested. From an economic point of view, hatchery manufacturers necessarily want to be able to detect as many duck eggs as possible at a time. Therefore, the higher the number of duck eggs in a single test, the better, as long as detection accuracy is ensured. Therefore, this paper tested several networks for duck egg detection on duck egg trays. All of them were based on the anchorless frame structure except the YOLOv4-Tiny and YOLOv5-S networks. Therefore, YOLOv4-Tiny and LDA models were used as examples, and the detection effects of the two networks are shown below. The duck egg images were divided into three sizes: 3 × 5, 5 × 7, and 7 × 9. The results show that without manually changing the YOLOv4-Tiny a priori frame hyperparameters, the anchor-frame-based YOLOv4-Tiny network could not accurately identify the duck eggs when the breeder egg image size changes, and the detection frame failed to fit the breeder egg. The YOLOX series network based on the anchor-free frame structure, on the other hand, did not have this problem. The detection results are shown in Figure 8.

## 4. Conclusions

In this paper, an infertility duck egg recognition algorithm based on the improved YOLOX-Tiny algorithm is proposed and implemented. Compared with existing detection models, the model in this paper has the advantages of low number of parameters and high detection accuracy. The algorithm proposed in this paper has three innovations: (1) The network is modified using deep separable convolution instead of the original convolution, and a new CSP structure and backbone network structure are proposed, which reduces the complexity of the backbone network and the size of the whole network. The number of model parameters is reduced from 5.03 M to 1.93 M and the mAP is increased from 94.92% to 99.74%. (2) The ECANet attention module is added after the backbone network, thus improving the detection accuracy of the network. (3) We use data augmentation and cosine annealing learning rate training techniques to avoid the model falling into local minima so that the network converges to the optimal solution. The experimental results show that the mAP of LDA model was 99.74%, the FPS was 57, the number of model parameters was only 1.93 M, and the FLOPs were only 4.66 G. Considering three aspects: detection accuracy, model size and FPS, the LDA model was found to be optimal. Therefore, this study provides a better method for identifying infertile duck eggs on incubation trays and provides a theoretical basis for improving hatching efficiency in the incubation industry.

## Figures and Tables

**Table 1:** The dataset of this paper.

Dataset	Fertilized Duck Eggs	Unfertilized Duck Eggs	Total
Training set	831	628	1459
Training set after data augmentation	3324	2512	5836
Validation set	220	106	326
Test set	220	106	326

**Table 2:** Improved network model size based on depth-wise separable convolution.

Models	Input Size (MB)	Parameters/M	FLOPs /G
YOLOX-Tiny	4.69	5.033	15.233
YOLOX-Tiny-DW	4.69	1.991	5.279

**Table 3:** Three different network model sizes based on CSPNet improvements.

Models	Conv	DWConv	Parameters/K	FLOPs /M
CSPNet	√	\	88.512	1145
	\	√	34.8	461.41
CSPNet_M	√	\	65.28	845.414
	\	√	29.472	389.53
CSPNet_S	√	\	42.048	545.587
	\	√	24.144	317.645

**Table 4:** Three backbone network size.

Models	Conv	DWConv	Parameters /M	FLOPs /G
YOLOX-Tiny	√	\	2.372	6.362
	\	√	1.024	2.671
LDA_M	√	\	2.246	5.205
	\	√	0.992	2.193
LDA_S	√	\	2.130	4.607
	\	√	0.967	2.056

**Table 5:** Initialization parameters.

Parameters	Value
Initial learning rate	0.001
Weight decay rate	0.0005
learning rate momentum	0.9
Number of iterations	600
Batch size	2
Optimizer	SGD

**Table 6:** Evaluation results of ablation experiments.

Models.	mAP/%	Parameters/M	FLOPs/G
YOLOX-Tiny	94.92	5.033	15.233
YOLOX-Tiny + DW	94.44	1.991	5.279
LDA_M	98.12	1.960	4.802
LDA_S	99.58	1.935	4.664
YOLOX-Tiny + ECA	95.16	5.033	15.235
YOLOX-Tiny + DW + ECA	94.65	1.991	5.282
LDA_M + ECA	98.30	1.960	4.804
LDA_S + ECA	99.74	1.935	4.667

**Table 7:** One-way ANOVA with mAP for different improvement methods.

Models	Models	*p*-Value
YOLOX-Tiny	LDA_S	0.000
YOLOX-Tiny + DW	LDA_M	0.000
LDA_M	LDA_S	0.000
LDA_S	LDA_S + ECA	0.001

**Table 8:** Comparison of model performance results of different feature extraction networks.

Models	AP/%		mAP/%	FPS	Parameters /M	FLOPs /G
	Fertile Egg	Infertile Egg				
YOLOv4_Tiny	84.35	60.75	72.55	141	5.87	16.17
YOLOv5_S	94.67	94.03	94.35	60	7.27	17.15
YOLOX_Tiny	99.84	90.00	94.92	57	5.03	15.23
LDA_M	99.69	96.90	98.30	58	1.96	4.80
LDA	99.91	99.57	99.74	57	1.93	4.66
YOLOX_Nano	99.65	88.88	94.27	56	0.90	2.55

**Table 9:** One-way ANOVA with mAP for different models.

Models	Models	*p*-Value
LDA	YOLOv4_Tiny	0.000
LDA	YOLOv5_S	0.000
LDA	YOLOX_Tiny	0.000
LDA	LDA_M	0.000
LDA	YOLOX_Nano	0.000

## Data Availability

The data presented in this study are available on request from the corresponding author.
